# Associations of Volatile Compounds with Sensory Aroma and Flavor: The Complex Nature of Flavor

**DOI:** 10.3390/molecules18054887

**Published:** 2013-04-25

**Authors:** Edgar Chambers, Kadri Koppel

**Affiliations:** The Sensory Analysis Center, Justin Hall-Dept HN, Kansas State University, Manhattan, KS 66506-1407, USA; E-Mail: kadri@ksu.edu

**Keywords:** flavor, aroma, sensory analysis, instrumental

## Abstract

Attempts to relate sensory analysis data to specific chemicals such as volatile compounds have been frequent. Often these associations are difficult to interpret or are weak in nature. Although some difficulties may relate to the methods used, the difficulties also result from the complex nature of flavor. For example, there are multiple volatiles responsible for a flavor sensation, combinations of volatiles yield different flavors than those expected from individual compounds, and the differences in perception of volatiles in different matrices. This review identifies some of the reasons sensory analysis and instrumental measurements result in poor associations and suggests issues that need to be addressed in future research for better understanding of the relationships of flavor/aroma phenomena and chemical composition.

## 1. Introduction

Flavor analysis using a variety of methods has been conducted for many years to help in the development of new products, to understand the nature of existing products, to study shelf-life, and to maintain quality of foods, beverages, products for oral care, and other products such as oral pharmaceuticals and tobacco [1,2]. Flavor analysis usually takes one of two forms, sensory or instrumental. Sensory descriptive methods used for testing have been developed that are highly reliable and consistent and obviously identify the human perception of flavor. Sensory analysis is the preferred method for evaluation of odor [3]. However, sensory methods are sometimes expensive to implement, may be time consuming when used properly, and sometimes cannot be implemented “on-line” for immediate feedback.

Instrumental methods for examining flavor also have developed that can provide feedback about the individual compounds associated with flavors. Those methods take many forms, but all are based on separation, identification, and quantification of compounds either in headspace or the actual product matrix [4]. These methods are particularly good at finding errant compounds, identification of compounds that may result in flavor changes, and when validated, some instrumental methods can be implemented to run continually in order to provide immediate or near immediate information about products.

Many studies have been published to understand the chemical composition of products. For example, numerous studies on wine composition have been conducted [5,6,7,8] to understand composition and lead to understanding specific sensory aspects of wine. A number of studies have been published evaluating effects of varietal differences and agricultural practices on foods [9,10,11]. Similarly, flavor compounds in other new or traditional foods frequently are examined to help better understand the product [12,13,14]. In other cases authors have tracked composition during manufacturing, storage or shelf-life [15,16,17,18].

Several review papers have addressed sensory-instrumental relationships or sensory interactions. For example, Poinot *et al.* reviewed methods that have been used to analyze aroma-related interactions [19]; Ross reviewed the human-machine interface in sensory science examining texture, sound, aroma, and flavor [20]; Croissant *et al.* reviewed sensory and instrumental volatile analyses applications of dairy products [21], and Auvray and Spence reviewed multisensory interactions between taste, smell, and the trigeminal system [22]. This review addresses issues in associating instrumental and sensory measurements, especially those intended to “predict” flavor based on chemical composition.

## 2. Flavor Measurement

### 2.1. Sensory Analysis

The primary measurement for the sensory aspects of flavor or aroma is descriptive sensory analysis, typically with trained sensory panels. Although there are many methods for conducting such analyses the methods typically examine the sensory perceived attributes and measure the intensities of those attributes [23]. Training of panels varies from a few hours for measurement of certain key attributes to months of training that may be needed to consistently measure nuances in flavor differences among products [24].

Of particular importance in measuring sensory aspects of flavor is the “naming” of attributes. This is important in order for multiple researchers to have a basis for understanding the product. Most techniques for developing attributes use some sort of definition or character referencing for individual attributes. A number of such sensory “lexicons” have been published recently, including ones for meat [25,26]; fruit and vegetable plants and products [27,28,29,30]; nuts and nut products [31,32,33]; beverages [34]; grain and grain products [35,36,37]; and dog food [38]. The use of carefully crafted lexicons is important when trying to compare to chemical data because using a general language may create confusion. Imagine, for example, trying to relate specific compounds in butter to a general sensory term “butter-like”. There is no single compound that could possibly be used to mimic the flavor of butter. Rather, more specific sensory characteristics such as dairy, waxy, fatty, coconut-like, rancid, or papery (to name a few) are needed to sensorially describe butter character.

### 2.2. Instrumental Flavor Analysis

In foods and beverages, headspace analysis is one of the options for instrumental determination of volatile compounds in a sample as the headspace contains all the volatiles that are responsible for the odor sensation. There are several options to isolate and concentrate the volatile compounds from the matrix, such as steam distillation/extraction or supercritical CO_2_ extraction [4] or the solid phase microextraction (SPME) [39]. Two common methods in instrumental volatile compound measurement are gas chromatograph-mass spectrometer (GC-MS) and a GC-MS coupled with an olfactometric port or a sniff port (GC-O) [1]. The GC-MS combines two techniques: a gas chromatograph to separate out the volatiles mixture in a sample and a mass-spectrometer to characterize each of the components individually. If this system is additionally equipped with a sniff port (GC-O) it is possible for a human to detect the compounds in the volatile compounds mixture that actually have an odor and therefore may be important in the sensory flavor of the sample.

GC-O methods are classified as detection frequency, dilution to threshold, or direct intensity [21]. Croissant *et al*. [21] has reviewed common methods for GC-O include aroma extract dilution analysis (AEDA), postpeak sniffing, combined hedonic aroma response measurements (CHARM), Osme, and nasal impact frequency/surface nasal impact frequency (NIF/SNIF). These studies often are followed by reconstitution studies of key compounds detected using sensory analysis [40]. Other instrumental flavor research methods with a focus on MS methods have been reviewed by Careri *et al.* [41]. More recent methods include recombining selected single compounds after eluting from the column into a mixture for sensory analysis [42].

In addition, it is sometimes possible to use an “electronic nose” to assess the composition of the volatiles compounds of a sample [43,44] and determine whether those compounds match predetermined groupings to identify products that may meet certain criteria. An electronic nose is composed of a number of sensors that interact with the volatiles that result in a change in their properties that is recorded and afterwards analyzed [45]. Electronic noses do not attempt to identify individual compounds and thus are more of an additional tool to GC techniques and sensory analysis.

## 3. Relating Sensory and Instrumental Methods—Why and How

Often, partial or full comparison of sensory testing with instrumental measurements is considered. This may be done when sensory testing takes up a lot of time from the judges and thus proves expensive, but also when there is a sound relationship established with sensory characteristics and instrumental measurements. In addition for some samples, such as wine vinegar, or other foods that have intensive aroma and flavor characteristics, instrumental aroma analysis may prove more practical [46], especially when frequent testing is needed. According to Lawless and Heymann [1] machines could be used instead of human judges in the following scenarios: (a) a correlation between a sensory characteristic and an instrumental measurement has been established, (b) there is a possibility that the sensory test is laborious and may damage the panelists’ health, and (c) the testing does not result in critical product-related decisions. The latter indicates that even if there is a proven relationship, sensory testing cannot completely be replaced by machines.

### 3.1. Direct Relationships

In a product development or benchmarking situation it may be that instrumental measurements are coupled with sensory analysis techniques to try and determine the exact volatile(s) responsible for some flavor sensations. This approach may prove helpful if certain aromatics in the product need to be enhanced or removed or there is a need to create further understanding of process-related aromatics.

It is possible to determine direct relationships between a sample odor and a chemical. Two common ways to do this are through either (1) comparative sensory analysis of the sample and the volatile compounds, using sensory analysis to detect aroma attributes and a GC-MS to detect the volatiles and find statistical associations, or (2) using humans sniffing at GC-MS ports to detect and identify and then sensors and computer programs to verify the compounds. In addition it is possible to calculate relationships, such as linear or non-linear correlations and multivariate regressions.

Direct relationships may seem like the easiest way to identify an odor compound by comparing the sample odor to a number of volatile compounds that may have a similar odor based on literature. This method does not require any instruments, but requires some knowledge of odorous volatile compounds. However, this method may prove to be laborious and yield only approximate conclusions as several chemicals may have identical odor characteristics, but different characteristics of the odor may appear at different concentrations of the chemical. This suggests instrumental measurements may prove useful if exact compounds need to be determined.

GC-MS may be used in combination with the sensory aromatic profile analysis to define the volatile compounds present in the sample. The main drawback of this method is that no information regarding actual aroma of a specific compound is acquired and thus false conclusions may be drawn when several chemicals change similarly to the associated sensory intensity. Additional literature research and attention to specific compound odor thresholds is also needed.

GC-MS sniff ports may be used to identify volatile compounds that have an odor detectable by the human nose. These compounds can then be related to the sensory aromatic and the mass-spectral profile. There may be some issues with actual perceived aroma, as compounds that may be sensed through the sniff port may be overwhelmed by other volatile compounds in the actual aroma of the sample. In addition sniff port measurements need to be replicated and conducted by several panelists as is done in sensory analysis. The sniff ports however may result in contradictory results as the flow of the compounds does not take into account the breathing of the panelists. This potential loss in detection capability should be overcome by careful sampling as well as the chromatographic information provided by the GC.

### 3.2. Calculated Relationships: Correlation and Regression

In the case of independent variable correlation or a possible relationship between two variables, scatterplots may be used to determine linear, logarithmic, or other associations [2]. Most common correlation methods assume a straight line relationship and determine correlation coefficients by determining how close the data points are from a straight fit through the data [1,47]. The correlation coefficient values would lie between −1 and 1, where −1 would show an inverse relationship and 1 a direct linear relationship. One of the most commonly used correlation coefficients is Pearson’s correlation coefficient. In addition there are some commonly used multivariate techniques such as Principal Component Analysis (PCA) or cluster analysis that can help in identifying relationships among variables.

**Figure 1:**
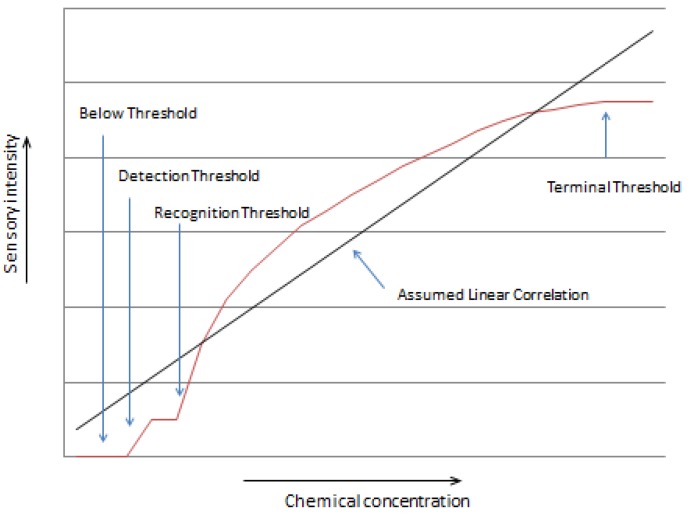
Schematic diagram of a potential relationship between amount of a chemical compound and sensory intensity showing various sensory thresholds. The linear relationship that was assumed is incorrect in this case.

One problem with using linear relationships is that sensory function and chemical composition are rarely linear. Often the relationships need to be converted using a logarithmic or other function. In many cases the sensory impact must reach a detection or recognition threshold, increases rapidly and then levels off as the chemical increases because a terminal sensory threshold is reached. In this case, the relationship often takes on a logarithmic function within the main sensory intensity of the relationship, but has no relationship before or after the detection or recognition threshold and the terminal threshold (see Figure 1).

In other cases, the actual perception may change as the chemical is increased. Various authors [48,49,50] have shown that as the concentration of chemical compound(s) increases, the sensory description can change. For example, pentane at low levels (1 ppm) was shown to be “beany”, at higher levels (10–5,000 ppm) it was “sweaty”, and at still higher levels (10,000–100,000 ppm) it had a barnyard/manure odor [49]. There is no statistic that can define such a relationship.

In the case of independent and dependent variables, regression analysis is used to predict the value of one variable based on the values of one or more other variables [2]. There are several techniques available. Partial least squares regression and General Procrustes Analysis (GPA) are commonly used to relate descriptive sensory and instrumental data. Partial least square regression (PLSR) is a multivariate statistical technique that has been proposed [51]. This technique has been used by several researchers for creating maps for determining relationships between instrumental volatile data (X-matrix) and descriptive sensory data (Y-matrix) [52,53]. GPA has been used to relate electronic nose data and sensory analysis data as well as GC-MS data with sensory analysis data [44]. However, the various computer programs used with these methods may produce different maps suggesting different relationships and further analysis often is necessary to determine actual relationships [54].

## 4. Examples of Identified Relationships and Potential Problems

### 4.1. Hexanal

The presence of hexanal is related to fat oxidation reactions in processed foods, but it is also present in a foods such as fruits and vegetables. Hexanal has been found in a variety of food products including meats and processed meats, fruits, processed fruits, as well as dairy and grain products. More specifically, hexanal often has been associated with green/grassy aromatics in fruits and vegetables. For example, a moderate correlation (0.56) between hexanal and the green/grassy attribute in tomatoeswas found [55]. In addition positive correlations have been found between green aromatics and an aldehyde mixture that contained hexanal used to spike tomato puree [56]; beany and grassy aromatics and hexanal and *trans*-2-hexanal in blackberries [57]; hexanal and green and leaf aromatics in soy milk [58]; hexanal and green/grassy aromatics in tangerines [59]; hexanal and green odorin cheese [60]; hexanal and a “lawn” attribute in olive oils [61]; and hexanal and green aromatics in strawberries [62]. However, [63] found no associations between hexanal and green tomato or grassy attributes in tomatoes.

In one study a negative correlation between hexanal and grass/sweet attribute was found, even though hexanal was used to train the sensory analysis panelists [44]. The authors suggested the panelists probably were not able to discriminate between aroma attributes for tomato cultivars. This indicates a need for well-trained panelists when correlations with volatile compounds are concerned. 

Some studies stated that hexanal had a green/grassy odor, but did not calculate correlations: black tea [64], hazelnuts [65,66], olive oil [67,68], artichokes [69], strawberries [70], mandarin peel [71], barramundi [72]; corn tortillas [73], pear juice [74]. Rather those authors indicated that according to previous studies hexanal should be associated with green/grassy aromatics. Stating such relationships is problematic because hexanal also is associated with other aroma/flavor characteristics.

In addition to green/grassy aromatics, in some foods hexanal often is associated with rancid and oxidized aromatics. For example Lee *et al.* found that hexanal is associated with rancid, acrid, and musty/earthy characteristics in black walnuts [52]. In addition Koppel *et al.* associated hexanal with oxidized oil aromatics in dry dog foods [53].

Other studies have found correlations between hexanal content and hay aromatics (0.58) and cheese aromatics (0.60) in honeys [75]. Whether these sensory attributes in fact could be related to hexanal by their description, is unclear, as attribute definitions or reference materials were not listed. Ercan *et al.* found hexanal in cheese to have woody aromatics, however, this was determined via GC-O sniffing port and not descriptive analysis [76]. Flores *et al.* associated hexanal with green/grassy aromatics from the literature, but with aromatics that contributed towards pork flavor in “Serrano” ham based on relationships in the study [77]. Forde *et al.* suggested hexanal in grapes is associated with peppery attribute in wines [78]. Krumbein *et al*. suggested hexanal is associated with moldy aromatics in tomatoes [79], while Limpawattana *et al.* suggested hexanal in glutinous rice had green tomato aromatics [80]. Maul *et al.* found a positive correlation between hexanal concentration and ripe tomato aromatics, sweetness (0.59), and tomato flavor (0.46), but no associations were found with green/grassy aromatics [43]. Mitchell *et al.* found that hexanal content is correlated with attributes such as salt flavor (0.75), yellow color (0.83), carrot aroma (0.81), overall flavor (0.83), overall flavor complexity (0.69), and aftertaste (0.70) in vegetable soups [81]. Those results indicate hexanal may have different characteristics depending on the concentration found and/or the flavor characteristics are actually a combination of several volatile compounds. In fact, Hongsoongnern and Chambers analyzed hexanal at different concentrations from 10 through 100,000 ppm and found that hexanal odor at low concentrations is musty/earthy (10–100 ppm), and at higher concentrations (5,000–100,000 ppm) has green-grassy/leafy, green-viney, musty-earthy, and pungent characteristics [48]. Vara-Ubol *et al.* found hexanal to have green/peapod, rancid, sour aromatics, and chemical-like aromatics [49], and Whitson *et al*. described hexanal as fatty/grassy [82].

One difficulty with volatiles odor characteristics is that mixtures of compounds may change human perception in various situations [83,84]. In fact, Kurin *et al.* stated that interactions produce unpredictable chemical activity [85]. That fact can explain much of the problem in identifying a single relationship to hexanal or other compounds. Bott and Chambers studied hexanal as part of a larger study to examine potential “beany” compounds [50]. Those authors noted that trained sensory panelists did not find that beany character in either hexanal or *trans*-2-nonanal when tested as single compounds. However, when combined at low levels of 10 ppm each, the combination became “beany”.

### 4.2. 3-Methyl-1-butanol

Some compounds, such as 3-methyl-1-butanol seem to have a variety of aroma characteristics associated with them.3-Methyl-1-butanol commonly is found in a number of food products and generally is considered a result of Strecker degradation or associated with lipid oxidation processes. Heil *et al*. related 3-methyl-1-butanol content to the alcoholic fermentation process and ethanol content (R^2^ = 0.86) in apple juices, but no correlation with sensory attributes were reported [86]. 3-Methyl-1-butanol has been associated with sensory attributes such as dark chocolate, pungent, and sweet in Turkish hazelnuts [66]. Costello *et al.* related 3-methylbutanol to harsh, nail polish, and herbaceous aromatics in wines [87], while Genovese *et al*. reported this compound to have green odor characteristics in wines [88]. Torrens *et al*. found 3-methyl-1-butanol to have alcohol and cheese odor characteristics according of the GC-O analysis of wines [89]. Ferreira *et al*. associated this volatile with fruity and alcohol aromatics in cheeses [90], but Ercan *et al*. found it to have bitter aromatics in Sepet cheeses according to GC-O analysis [76] and Moio *et al.* reported the compound to have fresh cheese odor according to the GC-O analysis [91]. Flores *et al.* found 3-methyl-1-butanol to have a penetrating green aroma in a GC-O analysis of Serrano ham, but did not find associations with sensory analysis characteristics [77]. In addition, Fukami *et al.* found 3-methyl-1-butanol to have a burnt aroma in a GC-O analysis of fish sauces [92]. Gomez Garcia-Carpintero *et al.* reported 3-methyl-1-butanol to have burnt and alcohol odor characteristics, but these were not related back to sensory attributes [93]. Karahadian *et al.* suggested 3-methyl-1-butanol may have malty odor in corn tortillas [73], while Garcia-Gonzales *et al.* reported this chemical to have spicy, malt, and burn odor according to GC-O analysis of olive paste [94].

3-Methyl-1-butanol has often been associated with other chemicals to contribute to a number of different aromatics. For example, this chemical in association with 2-methylpropanol was associated with balsamic-licorice sensory aromatics [95]. In addition 3-methyl-1-butanol has been associated with sunflower seed-like and nutty sensory attributes together with α-pinene and (*E*)-2-heptenal in sunflower seed oils [96]. Intentional mixing of 3-methyl-1-butanol and other chemicals resulted in beany, musty/earthy, musty/dusty, green/peapod, and nutty aromatics (hexanal), beany, musty/earthy, musty/dusty, green/peapod, nutty, and sour aromatics (1-octen-3-one), and beany, musty/earthy, musty/dusty, green/peapod, and floral aromatics (*trans,trans*-2,4-decadienal) [50]. Lee *et al.* reported that 3-methyl-1-butanol is one of the three major volatiles, together with 2-phenylethanol and diethyl succinate, that contribute to the base flavor of wines, but no correlations with the descriptive sensory analysis data were shown [97]. Niu *et al*. reported 3-methyl-1-butanol to have cheese odor according to GC-O, but associated this compound and furfural with the floral sensory attribute in cherry wines [98]. Guth reported that 3-methyl-1-butanol is an important odorant for white wine together with 2-methylbutyrate, 2-phenylethanol, 3-ethylphenol, and wine lactone, however, the author did not mention the specific odor characteristics [99]. Vilanova *et al*. found 3-methyl-1-butanol to contribute to aroma intensity together with hexanoic acid, octanoic acid, and phenylethyl acetate [100]. Jonsdottir *et al.* associated 3-methyl-1-butanol and 2-methylpropanal, 3-methylbutanal, and 3-hydroxy-2-butanone with sweet, flowery, caramel, melt-like odors and suggested these volatiles influence the flavor of ripened roe [101].

According to Costello *et al*. 3-methyl-1-butanol is a major contributor and flavor enhancer in wines, however there are few actual correlations with sensory attributes mentioned in the literature [87]. Vallverdu-Queralt *et al.* found a positive correlation with 3-methyl-1-butanol and sensory off-flavors and sweetness according to multivariate analysis, but did not report any numeric correlations in tomato juices [102]. Abegaz *et al*. found that in a correlation between instrumental volatiles and sensory attributes 2- and 3-methylbutanol correlated with the green/grassy attribute (0.46) and correlated negatively with fruity characteristics (−0.63) in tomatoes [55]. Hansen *et al.* studied aromatics in rye sourdough bread crumb and found that isoalcohols, including 3-methyl-1-butanol are important in the flavor of rye bread made with homofermentative cultures [103].

These findings indicate that there may not be definite associations between a chemical and an odor characteristic. Rather, this association changes according to the product matrix and composition.

## 5. Issues in Identifying Relationships

### 5.1. Poor Measurement and Identification

Ruth and O’Connor indicated that different GC-O methods do not necessarily yield a good correlation with sensory analysis methods [104]. This was demonstrated by the use of three GC-O methods, from which posterior intensity and detection frequency data correlated well with sensory analysis, while dilution analysis did not. In addition it was noticed that GC-O panel variation demands a panel size (n > 8) that may be unrealistic when using single sniff ports.

Although GC-MS techniques can be quite accurate, there are some issues. Heat labile compounds can be changed during the heating step of the GC. Also, it is also possible that the wrong compound is being identified during the analysis. When a number of compounds elute from the equipment at a similar time, it sometimes is difficult to conclusively identify a compound without further analysis. Any one of several compounds might be responsible for a particular odor note, but based on prior literature, a poorly trained technician chooses a particular compound as the “responsible” chemical without further checking.

Sensory testing also can be an issue. Sensory studies require the same care and measurement as chemical studies. Staff who clearly understand how to conduct research on flavor chemicals with instruments may have a poor understanding of the necessary training and standards needed to conduct high quality, replicable sensory research. It is imperative that well trained panels with considerable ability at identifying, naming, and quantifying sensory attributes be used in studies where chemical data will be related to the sensory data. Similarly, it is essential that in GC-O studies, panelists who are able to quickly and accurately describe/name sensory phenomena are sitting at the sniff ports.A lag of just a few seconds in identifying a sensory trait can cause the wrong compound to be paired with the aroma characteristic. Chambers *et al.* [24] and Otremba *et al*. [105] clearly showed that more training of sensory panelists resulted in better identification of attributes, less variation, and more ability to find differences in sensory aspects of samples. In addition to panelist issues, it may be that the human nose detects some compounds that were not detected by the instrument. For example, until the advent of more sensitive instrumentation in the 1990s, the “skunkiness” in light-struck beer was only able to be measured reliably using sensory methods.

### 5.2. “Noise” from Other Compounds and Attributes

Information from several GC-O studies indicates the same compound may exhibit different qualities for the human nose. The reasons for this may lie in the basic mechanisms that are responsible for aromatic sensations, such as odor receptors, odor concentrations, and odor thresholds. Some compounds present in the product matrix may be detected by the same receptors, thus changing the odor quality; in other products compounds may be eluted in a GC shortly one after another, which may influence the odor quality as well. In addition sample matrix and sample preparation may affect odor quality.

Another key aspect is that subthreshold levels of one or more compounds may influence the perceived properties of other compounds. For example, Ito and Kubota showed that a subthreshold addition of 4-hexanolide to subthreshold levels of (*E*)-2-hexenyl hexanoate, (*Z*)-3-hexenol, and indole changed solutions of those compounds from odorless to “astringent” or “heavy” smelling [106]. Dalton *et al.* showed that this can occur even across modalities when taste compounds are combined with subthreshold levels of odorants, a perception can occur [107]. This cross-modal effect does not have to happen at the molecular level, it may actually occur physiologically in the brain [108].

The matrix effect may influence analysis of products. For some products the matrix may bind flavor or physically hold compounds while other matrices do not. Wilson and Brown showed that the strength of the food matrix had a profound impact on the perception of banana flavor [109]. When the matrix had increased strength and melting point there was a concomitant decrease in intensity and increase in the time the flavor was perceived in the mouth. In addition there is an added compound-compound interaction possible that may influence flavor perception. Salles *et al*. reviews information on the physical and physiological breakdown or food and the impact on flavor perceptions [110].

Of importance also is the actual physicochemical interaction of the food matrix with flavor compounds. Voilley and Lubbers [111] discuss the interaction of the matrix compounds with flavor compounds in wine. They showed that the impact of yeast cells, proteins, ethanol, and other compounds can change the volatility of the aroma compounds, which changes the perceived wine aroma. A more comprehensive review by Guichard [112] discusses food ingredients more generally in a wider range of products. That author discussed a broad range of possible effects such as binding and forming complexes with various components such as proteins or amylose, differential effects on the solubility and flavor release of classes of aromatic compounds by fat, and the physical diffusion of the aromatics related to viscosity. Using model systems to represent actual food products to determine relationships between chemical compounds and sensory properties can result in spurious relationships that do not exist in actual food products.

### 5.3. Overlap and Variable Naming of Sensory Terminology

Sensory terminology also can impact finding relationships. Many sensory terms appear to overlap each other and may not represent a single specific sensory phenomenon. For example, Miller *et al.* found multiple sensory terms associated with “nutty”, a common term in sensory description of products [31]. Similarly, “green” aroma is not the same across products and different chemical compounds relate to different aspects of “green” [48]. Thus, it is essential that the sensory staff and panel clearly define the sensory aspect being described by the “attribute” used in the description. In addition, that attribute should be as specific as possible to describe the sensory characteristic. If the sample is simply green, then a wider array of chemicals could be responsible than if it is possible to more specifically define the characteristic as “green-peapod” or “green-grassy”.

Clear definition and proper training of sensory panels also can reduce misunderstanding from the use of different terms to describe the same sensory phenomenon. Although it is unlikely that two different panels would use widely disparate terms to describe the same sensory impact, it is reasonable, for example, that the use of terms such as rancid butter, sweaty, vomit, aged, etc. might be used to describe the “butyric” character of some cheeses. Those terms may have been ones used by the researchers previously or determined by the panel, but in any case they may or may not mean the same thing. Good definition of sensory terms used in describing products will help identify those terms that are used similarly or when they mean different things. In the example of “butyric” character with cheese, a highly trained panel would discriminate those terms easily. However, aless trained panel or a group of consumers might use those or a variety of other terms to describe the particular flavor in aged cheese. Unfortunately, the use of inexact terminology is a serious problem when trying to identify relationships between sensory attributes and chemical compounds. The use of “consumer-style” terminology (*i.e*., strong, smells like cheese, ripe, old, aged, *etc*.) may seem desirable at first glance as “real-life”, but such terminology is imprecise, can mean a variety of different things to different people, likely will not be highly related to any specific chemical compound, and often may not provide researchers or developers with the kind of data needed to solve a particular flavor issue.

### 5.4. Unexpected Relationships

One of the more difficult aspects of relating sensory characteristics to chemical compounds is when the compound is not “known” for having a particular character. Hexanal is “green” as most people describe it. However, some authors, as noted previously, describe it differently. These unexpected relationships may be real or anomalies of the analysis or may becaused by poor sensory panel training, but they always raise questions from scientists about the validity of the relationship. This is appropriate, but should not necessarily result in a flat dismissal of results. Certainly, more work is needed to determine if the relationship is real and the result of interactions with other aroma compounds, other matrix compounds, or is a hear-to-fore unidentified actual aroma/flavor resulting from that compound. 

### 5.5. Statistical Issues

One of the most important reasons that the relationship between chemical compounds and sensory perceived aromas and flavors is still difficult is the lack of direct linear relationships between the two. Most statistical applications, such as regression or even multivariate techniques such as principal components analysis use “linear” (including curvilinear) models to define relationships. This does not account for the fact that thresholds (detection, recognition, difference, and terminal) (see Figure 1) exist both for the chemical and sensory methods. This means that a chemical may need to reach a certain level before it can be measured or perceived and that at some point the sensory phenomenon may “max out” and no longer track with the physical intensity.

Statistics also rarely are able to examine multiple impacts and effects simultaneously in “real” systems without extensive testing. Conducting a multivariate analysis may point out possible relationships among multiple chemicals and multiple sensory attributes, but cannot predict cause and effect relationships without much more testing than usually is conducted in a single study. The advent of public databases will help with providing additional data for meta-analysis at some point in the future, but it is critical that the chemical and sensory analysis is conducted in the soundest way possible. Also, the composition matrix must be clearly defined in those databases because of the impact of various matrices on chemical interactions.

In addition, new statistical methods that rely less on linear assumptions and move into non-linear or so-called neural network processing are essential for better understanding of the relationships we want to understand. Possibilities include analyses such as logistic regression, which often is used to predict the presence or absence or some item, behavior, or action [113] and could be used to “predict” the likelihood that some sensory characteristics would be present given the presence of one or more compounds. However, this method does not provide a predictive equation of intensity to concentration. Furthermore, new statistical methods will be needed to account for alternative food matrices or to help explain and examine covariate relationships that may or may not impact the “structure-function” relationships that researchers want to discover related to chemical composition and the sensory perception of flavor.

One challenge in working with “predictive” or “associative” modeling is that the number of samples typically used in many studies is small. Particularly when multiple variables can be related, this small number of samples often makes it difficult to associate a single compound with a single sensory attribute, even if a relationship is known. Outliers (samples with a particularly different makeup or perception than other samples) create particular problems with small data sets because they can control what relationships are found or not [114]. At the same time, very large data sets may have so many “unique” products, chemicals, or attributes or too much “noise” in the data that it is impossible to determine associations because the sensory/chemical relationships were overwhelmed by other aspects.

Co-linear variables (ones that change similarly) also create problems in determining associations and these often occur in small data sets. Co-linear variables seem a though they are related even when they are not. For example, assume that both phenylethyl alcohol and hexanal change similarly among a small group of products—they are co-linear. We want to relate some compound to “rose” odor. The statistical modeling could actually show that hexanal is a better potential “predictor” of rose odor simply based on the similarity of the data. Of course in this case we know that phenylethyl alcohol is much more likely to be responsible for rose odor. However, in many cases when we begin to look for associations we often do not know what compound(s) may be responsible for various sensory properties.

## 6. Conclusions

This review identifies some of the reasons sensory analysis and instrumental measurements result in poor associations. Attempts to relate sensory data to volatile compounds have been frequent. Frequently, those associations have not been shown to be conclusive. The relationships often are difficult to interpret or are weak in nature. Occasionally, the methods used may not be as robust as needed to make the association, but difficulties also result from the complex nature of flavor. Multiple volatiles are responsible for a flavor sensation and although it is possible to pair some volatile compounds with some aroma or flavor sensations, this is not always the case. In complex products with complex flavors, combinations of volatiles may yield different flavors than those expected from individual compounds. The perception of volatiles in different matrices also may vary and rarely is accounted for when relating compounds with sensory phenomena over a range of products. We have suggested issues that need to be addressed in future research, for example improved analysis of data and meta-analyses, for better understanding of the relationships of flavor/aroma phenomena and chemical composition.
